# Effects of Exercise Continued Until Anaerobic Threshold on Balance Performance in Male Basketball Players

**DOI:** 10.2478/v10078-012-0046-0

**Published:** 2012-07-04

**Authors:** Nurtekin Erkmen, Sibel Suveren, Ahmet Salim Göktepe

**Affiliations:** 1 School of Physical Education and Sport, Selcuk University, Konya, Turkey.; 2 School of Physical Education and Sport, Gazi University, Ankara, Turkey.; 3 Department of Physical Medicine and Rehabilitation, Gulhane Military Medical Academy, Ankara, Turkey.

**Keywords:** balance control, basketball players, anaerobic threshold, stabilometer

## Abstract

The objective of the present study was to determine the effects of exercise continued until the anaerobic threshold on balance performance in basketball players. Twelve male basketball players (age = 20.92 ± 2.81 years, body height = 192.72 ± 7.61 cm, body mass = 88.09 ± 8.41 kg, training experience = 7.17 ± 3.10 years) volunteered to participate in this study. A Kinesthetic Ability Trainer (KAT 2000 stabilometer) was used to measure the balance performance. Balance tests consisted of static tests on dominant, nondominant and double leg stance. The Bruce Protocol was performed by means of a treadmill. The exercise protocol was terminated when the subject passed the anaerobic threshold. After the exercise protocol, balance measurements were immediately repeated. Statistical differences between pre and post-exercise for dominant, nondominant and double leg stance were determined by the paired samples t-test according to the results of the test of normality. The post-exercise balance score on the dominant leg was significantly higher than pre-exercise (t = −2.758, p < 0.05). No differences existed between pre- and post-exercise in the balance scores of the nondominant leg after the exercise protocol (t = 0.428, p > 0.05). A significant difference was found between pre and post-exercise balance scores in the double leg stance (t = −2.354, p < 0.05). The main finding of this study was that an incremental exercise continued until the anaerobic threshold decreased balance performance on the dominant leg in basketball players, but did not alter it in the nondominant leg.

## Introduction

In the present day, athletes are frequently exposed to knee and ankle injuries. Such injuries are widely seen in sports such as basketball, soccer, and volleyball. These injuries are usually revealed when athletes have direct contact or they occur during landing (Arendt et al., 1999; Gray et al., 1985). A successful landing involves strength, stability and balance (Devita and Skelly, 1992; Zhang et al., 2000). Therefore, most of the injuries result from a deficiency of strength or poor balance (Wikstrom et al., 2004).

In conjunction with increasing studies on postural control, it has been documented that balance has a high importance for athletic performance. The maintenance of balance has become necessary in order to prevent the injuries in both competitive sports and everyday life activities (Anderson and Behm, 2005). Postural sway and balance are denoted as an indicator of maintaining a stable posture (Lichtenstein et al., 1998). Postural control is an ability to stand with as little sway as possible (Gray et al., 1985). The ability to maintain balance demands the coordinated actuation of joint, muscle, visual and vestibular receptors (Ochsendorf et al., 2000). Fatigue or injury may negatively affect the sensorimotor system by restricting neuromuscular control and leading to a loss of balance (Tripp et al., 2007). Poor balance is characterized as a risk factor for ankle injury in basketball (McGuine et al., 2000) and soccer (Troop et al., 1984).

To assess the physiological state of the metabolic and respiratory systems, maximal oxygen uptake and ventilatory threshold measurements are often used (Wasserman et al., 1987). The energy needed at the beginning of an exercise is supplied by the anaerobic energy system, and then, aerobic metabolism meets the energy demand of the muscles (Tarnopolsky, 2004). The anaerobic energy system overrules when exercise intensity passes a certain level. After a short period of time, exercise intensity decreases largely due to the accumulation of H+ ions and metabolic acidosis (Janssen, 2001). The anaerobic threshold is characterized as the highest exercise intensity at which lactate is produced and is diffused at the same rate. Through anaerobic threshold which is an indicator of metabolic process, exercise intensity is parallel with heart rate, oxygen uptake and lactate level during an incremental exercise (Hanon et al., 1998).

Fatigue has adverse effects on neuromuscular control (Watson et al., 1984; Yeung et al., 1999). Muscle fatigue impairs proprioceptive acuteness due to the increase in muscle spindle discharge and disrupting afferent feedback (Pedersen et al., 1999).

In the studies examining the effects of exercise on postural control, authors have suggested that fatiguing exercise has an adverse effect on balance performance (Ageberg et al., 2003; Khanna et al., 2008; Gribble and Hertel, 2004; Nardone et al., 1997; Pendergrass et al., 2003; Springer and Pincivero, 2009; Wilkins et al., 2004; Yaggie and McGregor, 2002). Nardone et al. (1997) and Ageberg et al. (2003) postulated that the ability to maintain balance decreases after exercise compared to pre-exercise. Khanna et al. (2008), Susco et al. (2004) and Wilkins et al. (2004) reported that balance performance measured by the Balance Error Scoring System (BESS) decreases after fatiguing exercise.

The objective of this study was to determine the effects of exercise continued until the anaerobic threshold on balance performance in basketball players.

## Material and Methods

### Subjects

Twelve male basketball players (age = 20.92 ± 2.81 years, body height = 192.72 ± 7.61 cm, body mass = 88.09 ± 8.41 kg, training experience = 7.17 ± 3.10 years) from the basketball team at Gazi University volunteered to participate in this study. These participants were free from lower extremity injury, central nervous system injury, and any disorder that might affect neuromuscular control. Individuals read and signed an informed consent form approved by the local Ethics Committee.

### Balance assessment

A Kinesthetic Ability Trainer (KAT 2000, OEM Medical, Carlsbad, USA) stabilometer was used to evaluate balance. A Balance Index (BI) obtained by this device quantifies a person’s ability to keep the platform near the reference positions which are fixed before each balance test. An increase in the BI means a poor balance performance. Before the balance measurements, the psi value of the device was set to 6.0 to replicate the stance on a hard surface. Balance tests consisted of static tests on the dominant and nondominant leg, as well as the double leg stance. The dominant leg was defined by asking the person which leg he/she would preferably kick a ball with (11 right-legged and 1 left-legged persons). To become accustomed to the KAT 2000, subjects were allowed to familiarize themselves with the measurement device for 5 minutes before the pre-test. When the subject found the best position of balance, this position was recorded and used for all balance tests.

During the one-leg static test, the subject held the other knee at approximately 20° of flexion. In the double-leg static test, the subject stood on the platform in a comfortable position on either leg. During the measurements, the subjects stood with their arms crossed against the chest. The subject stood as quietly as possible in the test positions and tried not to grab the handrail with hands or legs. During all tests, the subjects’ positions were visually assessed by the physiotherapist. Before each measurement which lasted 20 s, the subject looked at the computer screen while maintaining a stable position. Then, the computer screen was turned away. During the balance tests, the subject looked at a fixed point. This point was located on the wall and in front of the KAT 2000, 180 cm above the floor and 130 cm in front of the center of the platform. When the subject grabbed the railing or could not maintain the test position, the measurement was canceled and repeated (Hansen et al., 2000).

### Exercise Protocol

Subjects were asked to refrain from exercise on the day of the evaluation. The exercise protocol was performed by means of a treadmill. The protocol began at a speed of 2.74 km/h and 10% gradient for 3 min using the Bruce protocol (Bruce, 1972). At the second stage, the gradient and speed were 2% and 4.02 km/h, respectively. In each subsequent stage of the test, the gradient and speed were increased in accordance with the protocol until each athlete exceeded their anaerobic threshold. During the exercise protocol, metabolic measurements of expired air were made with a Sensor Medics 2900-C metabolic gas analyser (SensorMedics 2900c, USA) and the *Breath by Breath* method was used. The expired air was connected to the system with a 2 way Rudolph Mask (Wasserman et al., 1987). The exercise protocol was terminated when the subject passed the anaerobic threshold (ventilatory threshold) level. The VO_2_ at the anaerobic threshold was measured by the V-slope method (Beaver et al., 1986). VO_2_, heart rate (HR) and exercise duration at the anaerobic threshold (ventilatory threshold) level were determined for each athlete. Participants performed the exercise protocol in running shoes on a treadmill, but all balance tests were performed in bare feet. After the exercise protocol, balance measurements were repeated immediately.

### Statistical Analysis

Descriptive statistics (mean ± SD) were calculated for all test variables. Statistical differences between pre and post-exercise for dominant, nondominant and double leg stance were determined by the paired samples t-test according to the results of the test of normality. Statistical significance was set at p < 0.05. Data were analyzed using SPSS software (version 11.0, SPSS Inc, Chicago, IL).

## Results

HR and VO_2_ at the anaerobic threshold measured during the exercise protocol are illustrated in Table 1.

**Figure 1:**
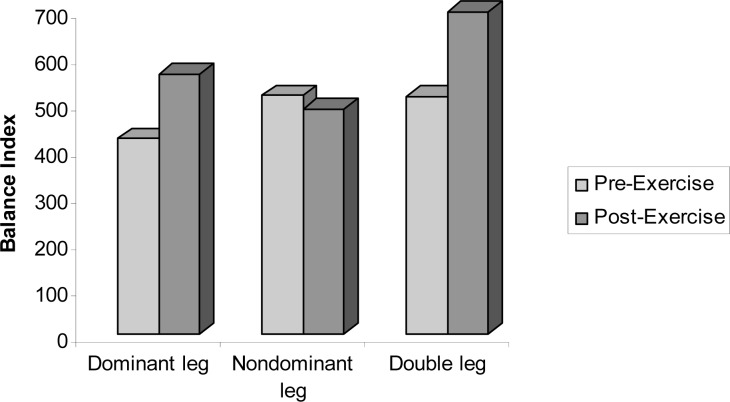
The balance index of basketball players in pre- and post-exercise

As shown in Table 2, the post-exercise balance score on the dominant leg was significantly higher than pre-exercise (t = −2.758, p < 0.05). The balance performance of basketball players on the dominant leg decreased after the exercise protocol. No differences existed between pre and post-exercise in the balance scores of the nondominant leg after the exercise protocol. A significant difference was found between pre and post-exercise balance scores in the double leg stance (t = −2.354, p < 0.05). After the exercise protocol, the balance performance of the double leg stance decreased, compared to pre-exercise values in male basketball players (Figure 1).

## Discussion

The purpose of the present study was to investigate the effects of exercise continued until the anaerobic threshold on balance performance in basketball players. The results of this study showed that an incremental exercise continued until the anaerobic threshold adversely affected balance performance on the dominant and double leg stance in male basketball players. On the other hand, the balance performance on the nondominant leg was not negatively affected by the exercise protocol.

Levels of blood lactate concentration increase during short term, high intensity exercise. In addition, oxygen uptake in the muscles is restricted due to exercise (Demura and Uchiyama, 2009). During aerobic exercise, the blood glucose level decreases due to the depletion of glycogen (Ishikawa and Takemiya, 1994). Physiological responses after aerobic and anaerobic exercises influence the postural performance in different ways (Demura and Uchiyama, 2009).

Many studies report that the ability to maintain balance is negatively affected by exercise (Ageberg et al., 2003; Gribble and Hertel, 2004; Khanna et al., 2008; Lepers et al., 1997; Nardone et al., 1997; Pendergrass et al., 2003; Springer and Pincivero, 2009; Susco et al., 2004; Waterman et al., 2004; Wilkins et al., 2004; Yaggie and McGregor, 2002). Agerberg et al. (2003) reported a significant increase in postural sway measured after short duration sub-maximal bicycle exercise. Lepers et al. (1997) noted that prolonged exercise decreases the ability to maintain balance in trained athletes. Nardone et al. (1997) reported that in maintaining balance, a decrease was observed with feet together after fatiguing exercise on a treadmill. Many authors have also indicated that the BESS scores increase significantly after fatiguing exercise (Khanna et al., 2008; Susco et al., 2004; Wilkins et al., 2004).

These studies suggest that postural sway increases after exercise. It is difficult to state definitely the postural performances between pre and post-exercise in this study because of the different types, duration and intensity of exercise and the physical characteristics of subjects. In this study, incremental exercise which was terminated after the subject passed the anaerobic threshold level was used as the exercise protocol. At the anaerobic threshold level, accumulation of lactate quickly increases in the blood and muscle cells (Janssen, 2001). Lactate accumulation in muscle cells provokes acidosis during exercise. Acidosis has an adverse effect on the mechanism of muscle contraction (Janssen, 2001). Khanna et al. (2008) used the Bruce Protocol as aerobic exercise stimuli to observe balance performance after exercise. In contrary to our study, the test was continued to exhaustion. They reported that balance performance decreased after this type of exercise.

Balance may vary according to sport disciplines of athletes, because each sport uses lower limbs differently. Basketball is a sport performed on land and players use their anti-gravity muscles extensively during training (Matsuda et al., 2008). Kellis et al. (2001) indicate that soccer players support their body mass with one leg while kicking the ball. In this study, balance performance significantly decreased on dominant and double leg stance after exercise, but did not change on the nondominant leg. Basketball players frequently perform jumping tasks during training and game situations. These jumps are performed as a last resort on the nondominant leg. The dominant leg is used less than the nondominant leg for jumping during a basketball match. Therefore, these findings suggest that the exercise does not have an adverse effect on balance performance of the nondominant leg. In parallel with the results of this study, Waterman et al. (2004) found that postural sway on the dominant leg increased after a game compared to pre-game values in female netball players, while postural sway on the nondominant leg was not different between pre and post game in these players.

The main result of this study was that incremental exercise continued until the anaerobic threshold decreased balance performance in the dominant leg in basketball players. A decreased ability to maintain balance may initially have a risk on the dominant leg. This risk can exist during both an exercise which is continued until anaerobic threshold and in high intensity anaerobic exercise. Basketball trainers and coaches should focus particularly on balance exercises for the dominant leg.

## Figures and Tables

**Table 1:** Summary data for physiological measures following exercise protocol

	***Mean***	***SD***
Exercise duration (min)	11.43	1.59
HR at anaerobic threshold (beat/min)	146.35	9.57
VO_2_ at anaerobic threshold (Lt/min)	1.75	0.43

**Table 2:** The means (± SD) and comparisons of the balance scores for pre-exercise and post-exercise

	***Pre-exercise***	***Post-exercise***	***t***	***P***
Dominant leg	422.33 ± 118.67	562.33 ± 224.36	−2.758	0.019^*^
Nondominant leg	516.25 ± 222.27	487.08 ± 155.88	0.428	0.677
Double leg	514.36 ± 238.73	697.09 ± 361.12	−2.354	0.040^*^

^*^ p<0.05
